# Feasibility, tolerance and effects of adding impact loading exercise to pulmonary rehabilitation in people with chronic obstructive pulmonary disease: study protocol for a pilot randomised controlled trial

**DOI:** 10.1186/s40814-021-00893-1

**Published:** 2021-08-03

**Authors:** Erin Cecins, Kylie Hill, Dennis R. Taaffe, David Manners, Anne-Marie Hill, Robert U. Newton, Daniel A. Galvão, Vinicius Cavalheri

**Affiliations:** 1 Curtin School of Allied Health, Faculty of Health Science, Curtin University, Perth, WA Australia; 2 Physiotherapy Department, St John of God Midland Hospital, Perth, WA Australia; 3 Institute for Respiratory Health, Perth, WA Australia; 4 Exercise Medicine Research Institute, Edith Cowan University, Perth, WA Australia; 5 School of Medical and Health Sciences, Edith Cowan University, Perth, WA Australia; 6 Respiratory Medicine, St John of God Midland Hospital, Perth, WA Australia; 7 School of Human Movement and Nutrition Sciences, University of Queensland, Brisbane, QLD Australia; 8 Allied Health, South Metropolitan Health Service, Perth, Australia

**Keywords:** COPD, Bone health, Impact loading, Treatable traits

## Abstract

**Background:**

Chronic obstructive pulmonary disease (COPD) is a disorder linked with a multitude of extra pulmonary manifestations (also known as treatable traits), including low bone mineral density (BMD). To date, no specific guidelines exist for the management of BMD in this population. Impact loading exercise has been identified as an intervention that improves or maintains BMD in other populations. However, the feasibility of and tolerance to impact loading exercise has not been tested in people with COPD. The aim of the proposed study will be to investigate the feasibility and tolerance of adding impact loading exercise to a standard pulmonary rehabilitation programme (PRP) in people with COPD and report its effects on bone health, balance and falls risk.

**Methods:**

This is a protocol for a pilot feasibility and tolerance randomised controlled trial (RCT). Fifty-eight people with COPD will be randomly allocated, on a 1:1 ratio, to either the experimental or control group. Initially, participants in both groups will complete a standard 8-week (twice-weekly) PRP followed by a 32-week period of maintenance exercises. Over the initial 8-week period, participants allocated to the experimental group will also undertake targeted lower limb resistance exercises and commence a programme of impact loading exercises (e.g. bounding and drop jumps). On completion of the initial 8-week PRP, in addition to the standard maintenance exercises, participants in the experimental group will continue with home-based impact loading exercises, four times a week, for the extra 32 weeks. The primary outcome of this study is feasibility of and tolerance to impact loading exercises. Feasibility will be measured using data collected pertaining to recruitment, withdrawal and completion. Adherence to the exercises will be collected using exercise logs. Tolerance to the exercises will be determined using outcomes to assess pain, recording any adverse effects such as a fall and feedback from the participants in semi-structured interviews on completing of the trial. The effects of the 40-week experimental intervention on bone health, balance and falls risk will be reported.

**Discussion:**

This pilot RCT will test the feasibility and tolerance of an intervention that has never been trialed in people with COPD. It will also provide initial information regarding the size of the effect this intervention has on outcomes such as BMD, balance and falls risk. These data will be critical when designing a definitive RCT to advance this area of research.

**Trial registration:**

Australian and New Zealand Clinical Trials Registry (ANZCTR): 12620001085965 (20/10/2020)

## Background

Chronic obstructive pulmonary disease (COPD) of moderate severity affects 1 in 10 adults over the age of 40 years and is projected to be the third leading cause of death globally by 2030 [1]. This disease is characterised by airflow limitation that is not fully reversible and results in persistent respiratory symptoms such as dyspnea [2]. However, it is now accepted that COPD is a systemic condition with several important comorbidities and extrapulmonary manifestations [2]. Of note, people with COPD die with their disease, not from their disease and comorbidities, and extrapulmonary manifestations are known to impact prognosis [3]. Optimal management of these comorbidities and extrapulmonary manifestations is now, in the era of precision medicine, recognised as a priority in the national and international guidelines for care of people with this condition [2, 4]. More recently, comorbidities and extrapulmonary manifestations in people with COPD have been badged as “treatable traits” [5].

Earlier work on the management of treatable traits in people with COPD has focused on interventions to ameliorate skeletal muscle dysfunction, obesity and anxiety and depression [6–8]. However, there has been little focus on the prevention of bone mineral density (BMD) loss in this population. Of note, people with COPD have greater loss of BMD when compared with the general population, as previous work has reported that as many as 70% of people with severe COPD have evidence of osteoporosis [9, 10]. This is considerably greater than the estimated prevalence in Australian adults aged ≥ 50 years of 9.4% (95% confidence interval [CI] 8.7 to 10.1%) [11]. This increased prevalence is concordant with the constellation of risk factors for loss of BMD that are frequently observed in people with COPD namely, older age, sedentary lifestyle, prolonged smoking history, low fat-free mass, prolonged use of inhaled corticosteroids and frequent short-term use of oral corticosteroids [12, 13].

Low BMD and osteoporosis in people with COPD are of great concern as this population has deficits in balance, which are likely to increase their falls risk. For instance, the prevalence of falls reported in people with COPD over a 12-month period (40%) is higher than the prevalence of falls over the same time period in community-dwelling older adults (29 to 33%) [14–16]. A study in a large cohort of elderly women (*n* = 4050) found that, second to osteoarthritis, the highest number of falls was associated with a diagnosis of COPD [17]. Loss of BMD increases the likelihood of fractures as a consequence of a fall (i.e. minimal trauma fractures), which in people with COPD can be catastrophic. Specifically, a previous population-based cohort study that included 11,985 participants (771 of whom had a diagnosis of COPD) demonstrated that 1-year relative risk (RR) for mortality following hip fractures was 71% higher in people with COPD than in those without COPD (RR [95% CI] 1.71 [1.55 to 1.88]) [18]. The increased prevalence of low BMD in people with COPD, coupled with their increased risk of falls and mortality following hip fractures, creates an urgent need to explore strategies that may enhance BMD or prevent further loss of BMD in this population [19].

Management and prevention of BMD loss in the adult population consists of pharmacological interventions (e.g. bisphosphonates) and lifestyle modification such as increased participation in physical activity, smoking cessation, limiting alcohol intake to ≤ 2 units/day and ensuring adequate dietary calcium intake and vitamin D [20]. Recent national guidelines for the prevention and management of osteoporosis recommend that, in order to reduce the risk of falls and rate of injurious falls, older adults should engage in high to very high intensity resistance training, moderate to high intensity impact loading exercise and balance training [21, 22]. Pulmonary rehabilitation is recommended as part of standard management for individuals with COPD [23]. Generally, pulmonary rehabilitation programmes (PRPs) consist of supervised exercise training, disease-specific education, self-management strategies and psychosocial support [23]. There is robust evidence showing the positive effect PRPs have on health-related quality of life (HRQoL), symptom management and exercise tolerance [23, 24]. To date, current national and international guidelines for PRPs do not include recommendations for impact loading exercise or balance training. The exercise training programme included in PRPs across Australia focusses on aerobic exercises, such as ground-based walking and some functional resistance exercises [25]. Although the effect of such exercise training on BMD in people with COPD is unknown, it is likely to have little if any effect because, during walking, the ground reaction forces resulting from the lack of a flight phase (which would be seen in running/jumping tasks) is insufficient to produce an osteogenic effect [26].

Studies in adults and older adults have suggested that impact loading exercises are safe and osteogenic [27, 28]. In fact, impact loading exercise has recently been tested in older men with prostate cancer undergoing androgen deprivation therapy, who have a high prevalence of comorbidities [29, 30]. The results from these interventions indicate that impact loading and resistance exercise can be safely undertaken in older men with prostate cancer and helps preserve spine and hip BMD as well as enhancing overall musculoskeletal function [29, 30]. There is a need to explore the effect on BMD of adding impact loading exercise to pulmonary rehabilitation in people with COPD.

## Methods

### Study aim(s)

The aims of this study are, in people with COPD:

1. To examine the feasibility of and tolerance to adding impact loading exercise to a standard PRP.
2. To report the effects of adding impact loading exercise to a standard PRP on bone health, balance and falls risk.

### Study design and participants

**Fig. 1:**
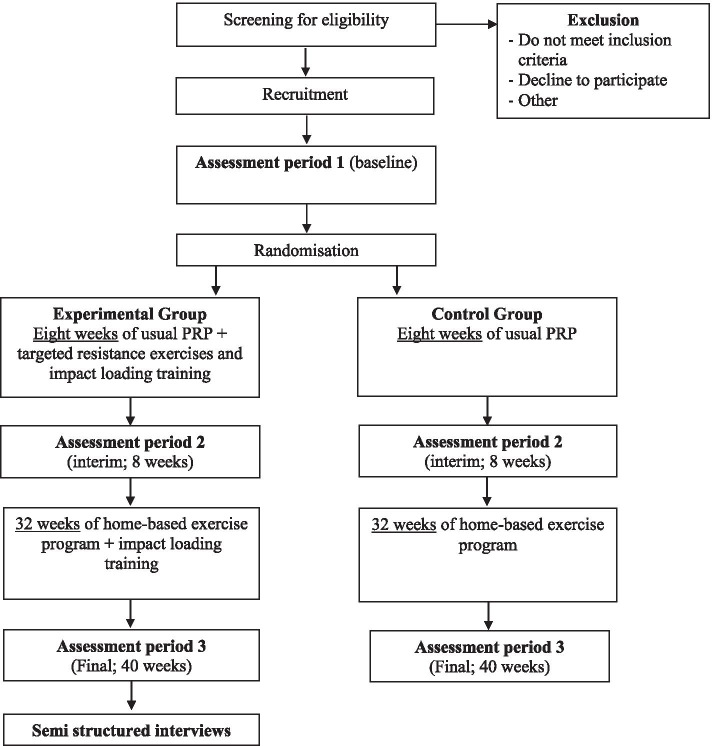
RCT protocol flow diagram

This is a protocol for a pilot, parallel group, feasibility and tolerance randomised controlled trial (RCT). The study has been approved by the ethics committees at St John of God Midland Hospital (#1494) and Curtin University (HRE2020-0044), in Perth, Western Australia, and has been registered with the Australian and New Zealand Clinical Trials Registry (ANZCTR) (12620001085965, 20/10/2020). It has been designed following the Standard Protocol Items: Recommendations for Interventional Trials (SPIRIT) checklist [31] and will be reported in accordance with the Consolidated Standards of Reporting Trials (CONSORT) extension for Pilot and Feasibility Trials statement [32]. The study flow diagram is presented in Fig. 1.

Patients will be deemed eligible for the study if they have a confirmed diagnosis of COPD [2] and have been referred to the PRP at St John of God Midland Hospital in Perth, Western Australia. People with COPD will be excluded if they (i) take prescription medication known to affect bone metabolism as the effect of the intervention on BMD will be unknown; (ii) have any mobility deficits, significant injury or surgical intervention that impairs their capacity to participate in weight bearing activity which is essential in order to complete the intervention; (iii) have any past or present evidence of a fragility fracture due to the risk of causing further harm; (iv) have any co-morbid conditions, physical or cognitive impairments thought to significantly compromise performance during the assessments and intervention; and (v) are unable to understand spoken or written English. Note: participants enrolled in the trial that are subsequently prescribed medication known to affect bone metabolism will continue in the trial and this information recorded.

### Recruitment and consent

Potential participants will be identified from the referrals received for the St John of God Midland Hospital PRP. If they meet the inclusion criteria, they will be invited to participate in the study by the primary investigator (EC). After written informed consent has been obtained, participants will complete baseline assessments and then be randomised into either the control or experimental group. The study intervention period will run over 40 weeks, with three assessment time points: (i) prior to randomisation (i.e. at baseline), (ii) on completion of the 8-week PRP (interim assessment) and (iii) on completion of the 40-week intervention period.

### Randomisation and allocation concealment

Participants will be randomly allocated on a 1:1 ratio. The primary investigator will be blinded to this process. The randomisation sequence will be computer-generated and stratified according to any moderate or severe exacerbations of COPD [2] within the past 6 months and BMD (i.e. *T*-score <  − 2.5 or *T*-score ≥  − 2.5) at either the spine or hip. The randomisation sequence will be generated and concealed using the Research Electronic Data Capture (REDCap) software [33].

### Pulmonary rehabilitation and home-based training programme (common to both groups)

The total duration of the intervention period will be 40 weeks. Participants in both groups will complete the standard 8-week PRP followed by 32 weeks of maintenance exercises to be completed at home. The PRP is undertaken twice a week and is supervised by a physiotherapist. It comprises all the core components recommended by the international guidelines [4, 23]. These include an aerobic component, most commonly a ground-based walking programme prescribed using results of a 6-min walk test (6MWT), functional upper and lower limb resistance exercises that require minimal equipment so they can be replicated at home (e.g. sit to stands), psychosocial support and disease-specific education which includes information on self-management strategies [23, 34]. Participants in the trial will receive group education sessions on the usual topics covered in PRP (i.e. benefits of physical activity) in addition to education specific for the individual (i.e. smoking cessation). To avoid contamination, participants in the experimental and control groups will attend the pulmonary rehabilitation classes on different days of the week. After completing the first 1 or 2 weeks of the PRP, participants in both groups will be encouraged to perform a home-based exercise programme. Whilst attending the PRP, participants will be instructed to complete this home programme on an additional 3 days each week, so that they are exercising 5 days a week (twice supervised, three times at home). On completion of the PRP, all participants will be instructed to continue with the home-based programme for 5 days per week as a maintenance programme. The home programme comprises a continuation of the exercises completed within the PRP sessions. In order to maximise musculoskeletal health and function, participants in both groups will be provided with calcium (1000 mg/day) and vitamin D (800 IU/day) supplements throughout the 40-week intervention period [21]. Each participant will be informed the reason why the supplements are being provided. Participants will be asked if they have previously taken or are currently taking calcium and vitamin D supplements. If they do, participants will be asked to cease taking their supplements and provided with the supplements for the trial. This will be undertaken in consultation with their General Practitioner (GP).

### Experimental intervention

In addition to the standard PRP, over the initial 8-week period, participants allocated to the experimental group will also undertake additional targeted, lower limb resistance exercises and commence a programme of impact loading exercise. The resistance exercises will be commenced at the beginning of the 8-week PRP and are designed to improve gluteal, hamstring, quadriceps and calf muscle strength in preparation for the impact loading exercises. The impact loading exercise will commence in week 5 of the PRP and will be undertaken in accordance with current recommendations on exercise prescription for the prevention and management of osteoporosis [21]. Details of the exercises including type, sets and repetitions are included in Table 1. The intensity, frequency, number of sets and repetitions of each impact loading exercise will be tailored taking into account the participant’s existing bone health status, co-morbidities and functional/clinical risk factors for falls and fractures. Each participant will be categorised as low, moderate or high risk for a fragility fracture and the prescription of the impact loading exercises will be modified accordingly [21]. The exercises will be modified as required, taking into account the participants physical capabilities and/or any reports of pain. All modifications to the exercises will be recorded in the exercise training logs.

**Table 1:** Progression and prescription of impact loading exercise

	**Phase 0**	**Block 1**	**Block 2**	**Block 3**	**Block 4**
**Weeks**	1–4	5–8	9–16	17–28	29–40
**Impact loading exercises**	N/A	- Bounding over soft hurdles (13–16 cm); 10 times - Drop Jumping (10–15 cm); 10 times	- Same as Block 1	- Bounding over soft hurdles (13–16 cm); 10 times - Drop Jumping (10–15 cm); 10 times - Hopping on one leg; 10 times	- Bounding over soft hurdles (13–16 cm); 10 times - Drop Jumping (10–15 cm); 10 times - Hopping on one leg; 10 times - Leaping; 10 times
**Number of rotation and rest between sets**	N/A	- Two rotations and 1–2-min rest between sets	- Three rotations and 1–2-min rest between sets	- Three rotations and 1–2-min rest between sets	- Three rotations and 1–2-min rest between sets
**Frequency**	N/A	- Weeks 5 and 6 twice weekly (supervised) - Weeks 7 and 8 twice weekly (supervised) and twice weekly (unsupervised)	- Four times weekly (home-based)	- Four times weekly (home-based)	- Four times weekly (home-based)
**Targeted lower-limb resistance exercises (In PR only, twice weekly)**	Calf raises, Knee flexion and extensions with multi gym, Seated hamstring curls with theraband if unable to complete knee flexion with multi gym, squats and lunges; 1–3 sets of 8–10 reps	- Same as phase 0	N/A	N/A	N/A
**Further Details**	Pulmonary rehabilitation only	- The first home-based session of this block will be supervised by a study investigator	- Home-based impact loading exercises will be the same as Block 1	- The first home-based session of this block will be supervised by a study investigator due to the progression of the impact loading exercise progression	- The first home-based session of this block will be supervised by a study investigator due to the progression of the impact loading exercise progression

The additional exercises will be closely supervised by a physiotherapist and implemented within the standard PRP using a circuit training approach. Those who tolerate the impact loading exercises well during weeks 5 and 6 of the PRP, with no reports of pain and no safety concerns, will be asked to complete an extra two sessions of home-based impact loading exercise in weeks 7 and 8. The first home-based session will be supervised by a physiotherapist. A suitable area will be established in the patient’s home environment to complete the exercises taking into account safety as well as a consistent floor surface. Participants will also be asked to wear the same or similar footwear every time they complete the exercises. In those who describe difficulties with the impact loading exercise in week 6 of the PRP, such as onset of pain or intolerable breathlessness, home-based training will be delayed until such time as they report no difficulties. Individuals who report issues with continence whilst performing the impact loading exercises will be offered a referral to a continence physiotherapy clinic. If difficulties during impact loading exercise are reported up to week 8, the individual’s participation in the intervention will be discontinued, but they will be asked to attend all remaining assessment sessions so their data can be analysed according to the intention to treat principle.

Following the completion of the 8-week PRP, in addition to the standard home exercise programme, participants who tolerate the impact loading will be instructed to continue with home-based impact loading exercise, four times a week, for an extra 32 weeks. In addition to the home visit scheduled during the PRP, in the 32 weeks following completion of the PRP, a physiotherapist will visit the participants at home on another two occasions to progress the impact loading exercises (Table 1). Participants will be given an additional exercise training log to track adherence and difficulties experienced with the impact loading programme.

Throughout the 32 weeks that follow the PRP, participants will be contacted once a fortnight by the primary investigator via phone to check their adherence to the exercises and discuss any difficulties or barriers they are experiencing with completing the programme, including any musculoskeletal issues such as pain. Throughout the intervention period, participants will be encouraged to contact the primary investigator with any adverse reactions or events. Specifically, this will include any falls, hospitalisations for any reason or visits to their GP for non-routine tests or appointments. In addition, participants will be asked about their adherence to taking the calcium and vitamin D supplements.

### Additional follow-up in control group

Following the completion of the 8-week PRP, participants in the control group will be contacted every 4 weeks, via a phone call, for 32 weeks. During the phone calls, they will be encouraged to report any issues with the home-based exercise programme and will be asked about any hospitalisations or visits to the GP for non-routine tests or appointments in the previous 4 weeks. In addition, participants will be asked about their adherence to taking the calcium and vitamin D supplements.

### Outcome measures

Measures related to the primary aim (i.e. feasibility and tolerance) will be recorded throughout the duration of the study using exercise logs and scheduled contact points with the participants. Assessments related to the secondary aim and those used to evaluate the PRP will be performed at three time points: (i) prior to randomisation (i.e. at baseline), (ii) on completion of the 8-week PRP (interim assessment) and (iii) on completion of the 40-week intervention period. During these assessment periods, measures will be made (across two non-consecutive days) of BMD, body composition, balance, falls risk, exercise capacity, HRQoL, functional limitation resulting from dyspnea and feelings of anxiety and depression. In addition, descriptive variables including height, weight, age, gender, lung volumes and smoking history will be recorded. Specific details on the measures and assessments are provided below. Body composition and BMD will only be measured at two time points, at baseline and on completion of the 40-week intervention period.

### Primary outcomes

#### Feasibility

To assess feasibility of the study protocol, data will be recorded pertaining to recruitment, withdrawal and completion, as well as adherence to the prescribed sessions (Table 2). The following cut-off points will be used to determine feasibility: recruitment rate of 50% (of participants screened as eligible for the study) and completion rate of 70% (including all assessments and intervention period). Adherence to all exercises prescribed during the PRP and at home will be tracked using exercise logs, completed by the participant. A completion rate of 75% of supervised and home-based exercises will be considered adherent to the intervention.

**Table 2:** Measures to assess feasibility of the impact loading exercise

Recruitment and completion	- Number of participants who were screened - Number of eligible participants who were recruited (i.e. provided written consent) - Number of participants who withdrew - Number of participants who completed the intervention period - Number of participants who completed all assessments and the intervention period
Adherence	- Number of supervised sessions completed - Number of home-based sessions completed

#### Tolerance

Tolerance of the experimental intervention will be compared to the standard PRP by:

1. Asking participants to rate any lower limb joint discomfort/pain at the completion of each exercise component of the exercise programme through use of the visual analogue scale (VAS; 0 to 10) [35]. These components include the walking programme, upper and lower limb resistance exercises and for the experimental group the targeted high-intensity lower limb resistance and impact loading exercises. During the home-based sessions, participants will be asked to record the VAS score for pain in a workbook and to contact the research team for guidance if they experience any increase in pain. Rating of perceived exertion (RPE) will be recorded using the RPE scale and breathlessness using the modified BORG dyspnoea scale [36].
2. Asking participants to immediately contact the primary investigator if they experience any adverse events, such as a fall, throughout the duration of the study. These data will be recorded by the primary investigator.
3. On study completion, feedback regarding their experiences will be sought from the participants, in the experimental group only, through semi-structured interviews.

### Secondary outcomes

#### Bone mineral density and body composition

Dual-energy X-ray absorptiometry (DXA, Lunar Prodigy) will be used to assess BMD (g/cm^2^) of the hip (total hip and femoral neck) and lumbar spine (L2 to L4) regions as well as whole body bone mineral content (BMC, g) and body composition. Regional and whole body lean mass (including appendicular skeletal muscle mass) and fat mass will be derived from the whole body DXA scan. The DXA scan will be conducted by a trained technician who will be blind to group allocation.

#### Balance

Balance will be assessed using the Berg Balance Scale (BBS) and the Four Square Step Test (FSST). The BBS is one of the most widely used clinical outcome measures to assess balance in older adults and has been shown to be sensitive to change following completion of a PRP in people with COPD [37, 38]. The FSST measures dynamic balance and is a reliable and valid measure for predicting falls in the elderly [39]. In addition the Activities-specific Balance Confidence (ABC) questionnaire will be used to determine participant’s confidence in performing activities without losing their balance and has good test–retest reliability and predictive capacity for falls in adults living in the community [40, 41].

#### Falls risk

Falls risk will be screened using the Falls Risk for Older People in the Community Screen (FROP-Com Screen). This is a 3-item falls risk screening tool that includes falls history in the past 12 months, functional status and balance. The score ranges between 0 and 9 and scores ≥ 4 indicate high risk of falls [42].

### Outcomes completed as part of the usual pulmonary rehabilitation programme

Other outcomes completed as part of the standard PRP will include the following: exercise capacity, measured using the 6-min walk test (6MWT) [43, 44]; HRQoL, measured using the interviewer administered version of the Chronic Respiratory Disease Questionnaire (CRDQ) [45]; functional limitation resulting from dyspnoea using the modified Medical Research Council Scale (mMRC) [46]; and anxiety and depression using the Hospital Anxiety and Depression Scale (HADS) [47, 48].

### Data management and analyses

Data will be entered and stored using REDCap data collection tools [33]. All data from the REDCap database will be de-identified. Statistical analyses will be performed using SPSS® (Statistical Package for Social Sciences, version 25.0 for Windows). As this is a pilot study, data pertaining to the primary aims (i.e. feasibility/tolerance) will be reported using descriptive statistics such as means and standard deviations (parametric data) or medians and interquartile ranges (non-parametric or ordinal data). Categorical data will be reported as frequencies and proportions. Estimates of the effect of the intervention on bone health, balance and falls risk will be determined using linear regression analysis and unstandardised beta coefficients.

### Sample size calculations

Justification of the sample size for pilot studies needs to balance issues of imprecision (small samples) against cost (large samples). As the primary goal of the pilot study is determining feasibility outcomes and not ensuring adequate power to detect a small between-group difference, it is inappropriate to calculate the sample size required to detect between-group differences. For this study, justification of the sample size aligns with an approach that has been described by experts that utilises confidence intervals (CI) [49]. The sample size will be large enough to provide a level of precision around an estimate of the effect for a given outcome (e.g. between-group difference in BMD) that would indicate that a definitive RCT in this area is worth pursuing. Rather than the usual 95% CI required for hypothesis testing, experts suggest that an 80% CI will satisfy the need for reasonable certainty for decision making based on pilot data. Using this approach, a sample size of 46 will allow us to comment on whether a standardised effect size for the change in BMD of 0.25 would be considered realistic in a definitive RCT. To account for a predicted 20% drop out rate, the target sample size for the study will be 58. In the previous annual report for 2017/2018, 222 people with COPD were referred to the pulmonary rehabilitation programme at St John of God Midland Hospital, a significant increase from the 138 patients referred the year before (2016/217). Of these, 81 were enrolled in the programme. We anticipate there will be a sufficient number of referrals to the programme to recruit 58 participants over a 2-year period.

## Discussion

The deterioration of bone health in people with COPD is of concern given that it is often undiagnosed until bone fractures occur. Recent studies have mainly focused on investigating the prevalence and risk factors for low BMD (i.e. osteoporosis) in people with COPD rather than developing strategies to ameliorate this problematic extrapulmonary manifestation of COPD.

This study will contribute novel information regarding the non-pharmacological management of bone health in people with COPD referred to a PRP. This pilot RCT will test an intervention, aimed at attenuating loss of BMD, that has never been trialed in people with COPD. It will investigate whether impact loading exercise, as a non-pharmacological intervention, is feasible and well-tolerated by this population. The pilot RCT will also provide initial information regarding the size of the effect this intervention has on outcomes such as BMD, balance and falls risk. These data are critical when designing a definitive RCT to advance this area of research.

## Data Availability

The full protocol, participant-level dataset and statistical code of the current study are available from the corresponding author on reasonable request.

## Abbreviations

ABC: Activities-specific Balance Confidence
ANZCTR: Australia New Zealand Clinical Trials Registry
BBS: Berg Balance Scale
BMC: Bone mineral content
BMD: Bone mineral density
CI: Confidence interval
CONSORT: Consolidated Standards of Reporting Trials
COPD: Chronic obstructive pulmonary disease
CRDQ: Chronic Respiratory Disease Questionnaire
DXA: Dual-energy X-ray absorptiometry
FROP-Com screen: Falls Risk for Older People in the Community Screen
FSST: Four square step test
GP: General Practitioner
HADS: Hospital Anxiety and Depression Scale
HRQoL: Health-related quality of life
mMRC: Modified Medical Research Council Scale
PRP: Pulmonary rehabilitation programme
RCT: Randomised controlled trial
REDCap: Research electronic data capture
RPE: Rate of perceived exertion
RR: Relative risk
SPIRIT: Standard Protocol Items: Recommendations for Interventional Trials
SPSS: Statistical Package for Social Sciences
VAS: Visual analogue scale
